# Experimental Evidence for Nutrition Regulated Stress Resistance in *Drosophila ananassae*

**DOI:** 10.1371/journal.pone.0046131

**Published:** 2012-10-01

**Authors:** Seema Sisodia, Bashisth N. Singh

**Affiliations:** Department of Zoology, Banaras Hindu University, Varanasi, Uttar Pradesh, India; University of Dayton, United States of America

## Abstract

**Background:**

The amount and quality of nutrients consumed by organisms have a strong impact on stress resistance, life-history traits and reproduction. The balance between energy acquisition and expenditure is crucial to the survival and reproductive success of animals. The ability of organisms to adjust their development, physiology or behavior in response to environmental conditions, called phenotypic plasticity, is a defining property of life. One of the most familiar and important examples of phenotypic plasticity is the response of stress tolerance and reproduction to changes in developmental nutrition. Larval nutrition may affect a range of different life-history traits as well as responses to environmental stress in adult.

**Principal Findings:**

Here we investigate the effect of larval nutrition on desiccation, starvation, chill-coma recovery, heat resistance as well as egg to adult viability, egg production and ovariole number in *Drosophila ananassae*. We raised larvae on either protein rich diet or carbohydrate rich diet. We found that flies consuming protein rich diet have higher desiccation and heat shock resistance whereas flies developed on carbohydrate rich diet have higher starvation and cold resistance. Egg production was higher in females developed on protein rich diet and we also found trade-off between egg production and Egg to adult viability of the flies. Viability was higher in carbohydrate rich diet. However, sex specific viability was found in different nutritional regimes. Higher Egg production might be due to higher ovariole number in females of protein rich diet.

**Conclusion:**

Thus, *Drosophila ananassae* adapts different stress tolerance and life-history strategies according to the quality of the available diet, which are correlated with phenotypic adjustment at anatomical and physiological levels.

## Introduction

The most obvious way by which environmental variation may influence body condition and fecundity is via nutritional effects resulting from variability in food type availability. In general terms, diet effect can be classified as either quantitative (i.e. food availability) or qualitative (i.e. food composition). The quantitative effects are evident since animals obtain energy and other nutritional requirements from food. Thus, under a natural range of conditions there is a positive correlation between food availability and body condition or fecundity. Qualitative effects often are divided into two categories: namely nutritional deficiencies and inhibitory metabolites.

The balance between energy intake and expenditure is necessary to the survival and reproductive success of animals [1], [2]. This balance depends on the interplay between matter intake, digestion and allocation of acquired energy to various functions such as maintenance, growth and reproduction [3]. Animals obtain energy and nutrients from food, so diet can be considered a key factor that potentially affects all life-history components [4], [5]. Experimental modifications of animal diets have played a key role in the study of how organisms adjust their energy allocation [6], [7].

The amount and quality of nutrients intake by organisms have a strong impact on life-history traits, such as disease vulnerability, fertility, reproduction, longevity and stress resistance [8], [9], [10]. Studies concern with the impact of nutrition often assesses the physiological and morphological responses of individuals exposed to different quality and amount of nutrients.

Many organisms face a challenge of meeting their optional nutritional requirement for somatic and reproductive growth under natural conditions [11]. During development, body tissues constantly require a specific quantity and proportion of nutrients in order to attain optimal growth and performance [12]. Deficiency or imbalance of fat, carbohydrate or protein can affect characters such as growth and reproduction. Protein deficiency reduces fecundity and growth in *Drosophila melanogaster* [13] and in fruit-feeders protein is often limiting macronutrients [14], [15], [16], [17], [18]. In contrast diet restriction on mild starvation can increase longevity as well as tolerance to stressors such as heat stress [19], [20] demonstrating the complexity of organismal nutrient acquisition and utilization. A variety of factors may affect organismal stress tolerance. These include physiological as well as behavioural changes. The bulk of studies on physiological and evolutionary responses to nutrient deficiencies focus on reproduction and fecundity [12], [17], [21], [22].

Biological stress may be defined in evolutionary terms [8]. Sibly and Calow [23] broadly define stress as an environmental condition that when first applied, impairs Darwinian fitness and similarly Koehn and Bayne [24] define stress as any environmental change that acts to reduce the fitness of an organism. Genetic variation in stress tolerance will result in adaptive change to an extent that depends on the frequency of environment faced by the organism and the associated physiological costs [8]. Unsuitable or insufficient food resources resulting in deprivation of normal nutrients constitutes environmental stress and it has been argued that stress associated with marginal resources impacts populations of most species [25]. Because stress resistance traits in *Drosophila* often vary across latitudinal clines [26], it is likely that selection affects resistance traits either directly or indirectly. Individuals within many species must survive periods of starvation or exposure to suboptimal diets. As a consequence, positive selection for resistance to starvation stress is expected in localities where food is likely to be less abundant or temporarily less reliable. When faced with nutritionally imbalanced diets, compensatory feeding for the limiting nutrients results in over ingestion of other nutrients, as is often seen when insects are confined to food low in protein relative to carbohydrate [11], this may result in increased lipid storage and reduced fitness [27], [28].

Organismal stress tolerance is affected by variety of factors. Climatic changes may be met by physiological hardening processes, coma or production of metabolites making the organism tolerate temperature extremes [29], [30], [31]. Also an organism may compensate for nutritional stress and reduced body size by extending its growth period or altering its energy allocation to growth, hence postponing the reproductive period [32], [33].

Fecundity (number of offspring produced) comprises one of the most energetically expensive processes involved in reproduction and usually is taken as a proxy value for the total reproductive efforts [34], [35]. For invertebrate animals changes in fecundity due to dietary effects have been recorded for different systems and taxa including changes associated with food limitation [36], [37], moisture content in the diet [38], specific nutrient deficiency [4], [39], diet composition [40], [41], [42] and presence of inhibitory secondary metabolites [43], [44].

*Drosophila ananassae*, a cosmopolitan and domestic species belonging to the *ananassae* subgroup of the *melanogaster* species group is stenothermic and circumtropical in distribution. India is a large tropical and subtropical continent and covers a large range of latitude and altitude. From south to north, the seasonal thermal amplitude shows a regular increase with progressively more marked cold and warm seasons. Seasonal variations strongly increase with latitude.

Sisodia and Singh [26], [45] found a high degree of variation in stress resistance at the population level in *Drosophila ananassae.* In India, feeding habit and composition of food vary with latitudes and obviously ratio of protein:carbohydrate also varies accordingly. Apart from clinal effects, the availability of food of that particular area is also an important factor for variation in stress resistance. Keeping this in view, the aim of the present study was to evaluate the physiological adjustments and the life-history effects of changes in energy allocation under different diet regimes. Thus we have investigated the relative importance of two important macronutrients on desiccation, starvation, thermal tolerance (hot and cold), life-history traits- egg to adult viability, egg production and morphometric trait- ovariole number. *Drosophila ananassae* occupies a unique status among the *Drosophila* species due to certain peculiarities in its genetic behaviour. It is of common occurrence in India. Behavioural studies also have revealed several interesting features in *Drosophila ananassae* [46], [47], [48]. Together with the fact that *Drosophila ananassae* has unique features, this makes this species ideal for experimental studies aiming at investigating how diet composition during larval stage of *Drosophila ananassae* shape the evolution of physiological stress, life-history traits and morphometric traits.

## Materials and Methods

### Stock Investigated

The RN stock of *Drosophila ananassae* used in the present experiment, was established from flies collected from fruit and vegetable baits in Ranchi (Latitude 23.21°N), Jharkhand, India in October 2010. For maintaining the stock, simple culture medium containing Agar-Agar, dried yeast, maize powder, crude sugar, nipagin, propionic acid and plain water were used. Fifteen pairs of flies were transferred to fresh culture bottle in each generation to maintain stocks. Prior to the experiment flies were kept in simple culture medium. Eggs were collected and transplanted to two types of food media: a carbohydrate enriched medium and protein enriched medium. Both media were made by mixing either sucrose or casein with simple culture medium. The carbohydrate-enriched medium (20% carbohydrate) was prepared by mixing sucrose and culture medium in 1∶4 ratio, before adding water. The protein enriched medium (60% protein) was prepared by mixing casein and simple culture medium in a 3∶2 ratio. All vials contained approximate 7 ml. of medium and pasted with dried yeast solution. In each vial, 20 eggs/vial were kept. The eggs hatched and larvae developed at 25°C and 12 h L/D cycles. Virgin flies were collected and aged for 6–7 days before starting the experiments. We followed the method of Andersen et al. [49].

### Desiccation Resistance

Kennington et al. [50] method was followed to measure desiccation resistance. Desiccation resistance was measured on 4–5 days old virgin flies, and up to 10 flies of each sex were measured for each population. Five replicates were carried out. To measure resistance, flies from each vial were transferred to new vial containing a disc of dry filter paper and covered with muslin gauze cloth secured with an elastic band. Desiccation vials were kept at 25°C under constant light and were observed for the number of dead flies seven hours after the flies were originally transferred and then at half-hourly intervals until all the flies had died.

### Starvation Resistance

Starvation resistance was measured on 4–5 days old virgin flies and, as with desiccation resistance, up to 10 flies of each sex were measured for each population. Five replicates were carried out. To measure starvation resistance, flies from each vial were transferred to a new vial containing 7 ml. of 1% agar and plugged with cotton in order to prevent desiccation. Starvation vials were kept at 25°C under constant light, and were observed for the number of dead flies 40 h. after the flies were originally transferred, and then at 6-hourly intervals until all the flies had died.

### Recovery Time (RT) from Cold Treatment

Flies were aged for 5 days and transferred without anesthesia in empty glass vials which were immediately kept at 5°C. 50 flies of each sex were taken for experiment. The duration of cold treatment was 16 h. For measuring recovery time adults were placed into a Petri dish at room temperature. At the beginning, all flies were in chill coma and unable to move. The transfer to room temperature permits a progressive recovery, starting by the capacity to move the tarsi, then the legs and finally to stand up. We considered a fly recovered from chill coma when it could stand on its legs, the fly was then recovered from the Petri dish, and the time changed from the beginning note as an estimate of RT. For each group, the mean RT was calculated and used as a basic observation. We have followed the methods of Ayrinhac et al. [51].

### Heat Shock Survival

Flies were heat-shocked in empty food vials. To prevent desiccation the stoppers were moistened with tap water. The vials were placed evenly spaced in racks in incubators. One group was hardened at 37°C temperature for 1 h; followed by 1 h at 25°C temperature to allow the flies to recover before being heat shocked 1 h at 40°C temperature. The other group was directly exposed to 40°C temperature for 1 h. In each group 10 flies per vial and five vials per sex and population were used. After the heat shock, flies were transferred to fresh food vials and allowed recovery for 24 hrs at 25°C temperature before survival (ability to walk) was scored.

### Egg to Adult Viability

Twenty female and twenty male flies from the mass population developing on simple food medium were collected. They were divided in four groups of 10 flies (5 males and 5 females in each group) and left to rest on standard food medium for 2 days. After 48 h flies were transferred to a new vial for egg laying on standard food medium during a period of 4 h. Eggs were collected and transferred in groups of 20 to vials containing 7 ml. of either carbohydrate-enriched medium or protein –enriched medium. Eggs were left to hatch at 25°C with 12 h light/12 h dark cycles. We checked for newly emerged flies every 8 h and counted and sexed the emerging flies.

### Egg Production

We collected virgin males and females developed on either the carbohydrate-enriched medium or the protein- enriched medium. One male and one female were transferred to vials containing standard food medium. Pairs of flies were transferred to vials with teaspoons with standard food medium at 3–4 days. Every 48 h flies were transferred to a new vial with new teaspoon with standard medium. This procedure was repeated three times so that the number of eggs produced from 3–4 days to day 7–8 of age could be registered.

### Counting of Ovariole Number

In females, the ovaries were dissected in insect saline (0.67% NaCl), stained with 2% acetocarmine stain, and mounted in 45% acetic acid; the ovariole number was counted under a microscope at 50x magnification.

### Statistical Analysis

In analyzing survival data, two functions that are dependent on time are of particular interest: the survival function and the hazard function. The survival function S (t) is defined as the probability of surviving at least to time t. the hazard function h(t) is the conditional probability of dying at time t having survived to that time. The graph of S (t) against t is called the survival curve. The Kaplan-Meir method was used to estimate this curve from the observed survival time without the assumption of an underlying probability distribution [52].

Comparison of two survival curves was done by using a statistical hypothesis test called the log rank test. It is used to test the null hypothesis that there is no difference between the population survival curves (i.e. the probability of an event occurring at any time point is the same for each population).

Two-way ANOVA was used to analyze egg to adult viability. Comparison of egg production and difference in ovariole number in females developed from two different nutritional regimes were analyzed through Student’s t-test.

## Results

### Desiccation Resistance

**Figure 1:**
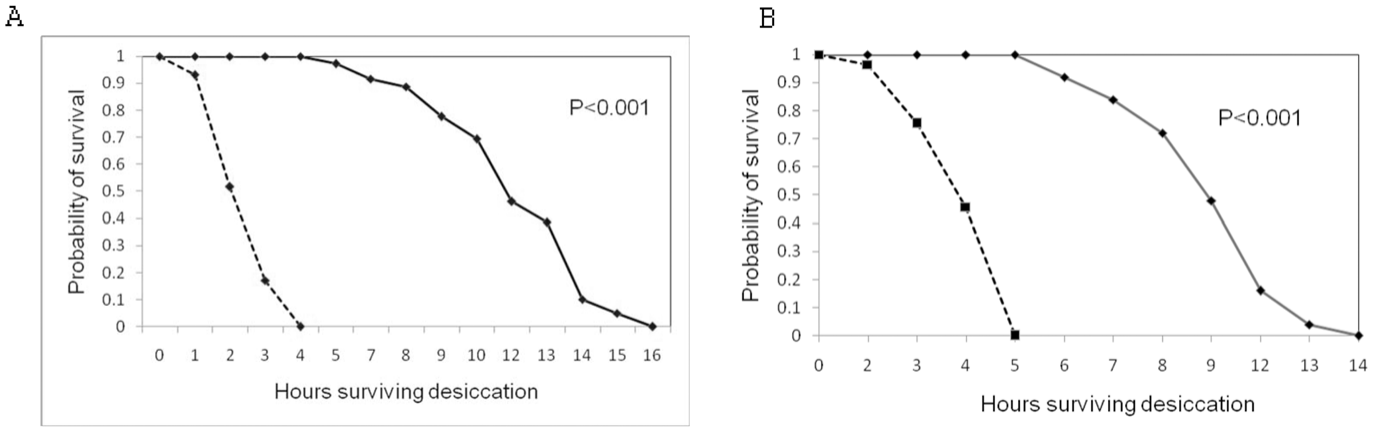
Survival curves for desiccation resistance in males (A) and females (B) derived from either protein (–) or carbohydrate (- - -) enriched medium.

Desiccation resistance was affected by nutritional regimes. Males and females flies developing on the protein enriched medium have higher desiccation resistance than flies developed on carbohydrate –enriched medium. There is highly significant variation in survival days of two types of flies in both the sexes (Fig. 1a, b).

### Starvation Resistance

**Figure 2:**
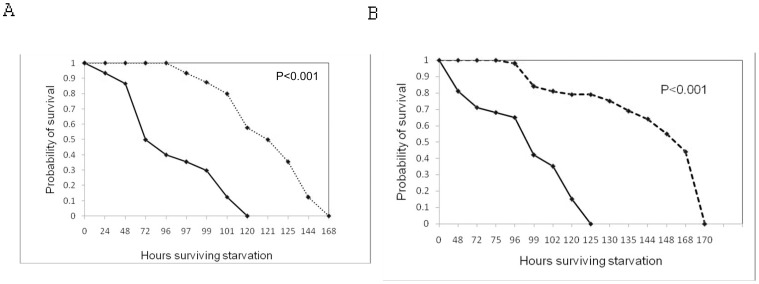
Survival curves for starvation resistance in males (A) and females (B) derived from either protein (–) or carbohydrate (- - -) enriched medium.

We observed higher starvation resistance in flies developed on protein enriched medium than flies developed on carbohydrate enriched medium. There is highly significant variation in survival days of two types of flies in both the sexes (Fig. 2a, b).

### Chill Coma Recovery

**Figure 3:**
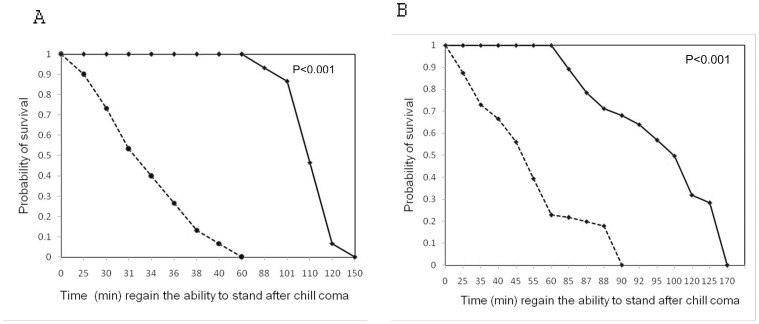
Survival curves for chill-coma recovery in males (A) and females (B) derived from either protein (–) or carbohydrate (- - -) enriched medium.

Chill-coma recovery time of the flies was significantly affected by nutritional regimes. Flies developed on protein-enriched medium recovered more slowly than flies developed on carbohydrate-enriched medium (Fig. 3a, b).

### Heat Shock Survival

**Figure 4:**
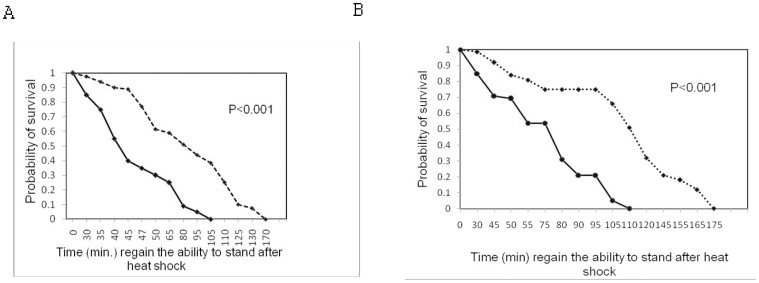
Survival curves for heat – shock in males (A) and females (B) derived from either protein (–) or carbohydrate (- - -) enriched medium.

We found a significant influence of nutritional regimes on heat shock survival. Flies developed on protein-enriched medium have fast recovery from heat shock than flies developed on carbohydrate –enriched medium (Fig. 4a, b).

### Egg to Adult Viability

**Figure 5:**
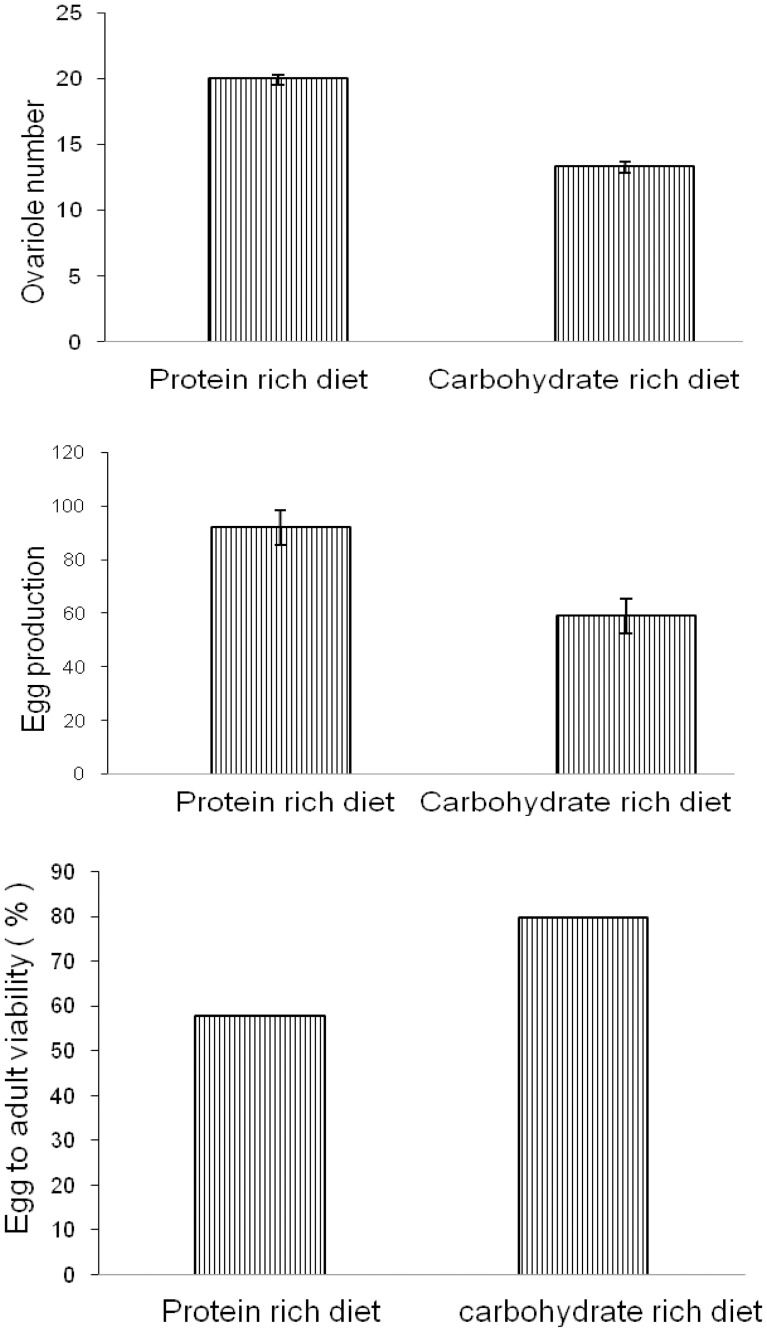
Ovariole number, egg production and egg to adult viability of flies developing on protein rich diet and carbohydrate rich diet. Bar represents Mean ±SE.

Assuming that the sex ratio of the eggs collected is 50∶50. Table 1 shows that there is a significant effect of nutritional regimes and sex on egg to adult viability. We also found a significant interaction between sex and nutritional regime on egg- to – adult viability. On average 20% more females developed on protein-enriched food, while on average 30% more males developed on carbohydrate-enriched medium. Viability is greater in carbohydrate rich diet (Fig. 5).

**Table 1:** Two – way ANOVA for egg to adult viability in either protein or carbohydrate-enriched medium.

Trait	Source	d f	F	P
Egg to adult viability	Fly nutrition	1	11.58	<0.001
	Fly sex	1	12.57	<0.001
	Fly nutrition x fly sex	1	9.25	<0.001
	Error	126		

### Egg-production and Ovariole Number

We found a significant difference in egg production of females developed on different nutritional regimes when tested on simple culture medium. (t-test: 6.57 p<0.001). Average female egg production/day is 63 in case of females developed on carbohydrate –enriched medium while protein fed female’s average egg production is 94. There is significant difference in ovariole number between females developed in two types of food (t-test: 5.24, p<0.001). Females developed on protein rich food have higher ovariole number than females developed on carbohydrate rich food. So there is positive correlation between number of eggs laid and ovariole number. We also found trade-offs between egg production, ovariole number and egg to adult viability (Fig. 5).

## Discussion

The findings of this study demonstrate the importance of food availability at the larval stage on ability of adult flies to cope with various types of stress. In this study, we found a clear evidence for impact of nutrition on the ability to cope with stress as well as on the life-history traits. As larval nutrition regulates the stress resistance, our results indicate the stress tolerance and nutrition are tightly linked. Given different viability in two types of medium, we cannot rule out that not only plastic response but also selection can be involved in explaining our results.

### Desiccation Resistance

Tolerance to desiccation stress was highest in flies developed on the protein enriched medium. Our results support the findings of Anderson et al. [49], where *Drosophila melanogaster* flies developed on protein diet show higher survival in desiccation stress. Our findings also strongly support our previous report [26] in which south Indian populations of *Drosophila ananassae* mostly feed on carbohydrate rich food and have low desiccation stress as compared to north Indian populations which feed on protein rich diet. The composition of food may vary with latitude and altitude of origin of population too. The possible explanation for high desiccation tolerance is that the metabolic end product from protein metabolization, uric acid had a protecting effect on the increasing osmotic pressure during desiccation by reducing the water loss from cells [53].

### Starvation Resistance

As far as our information goes, this is first report on comparison of starvation resistance of any *Drosophila* species that develop on either protein or carbohydrate-enriched medium. In our study flies developed on carbohydrate enriched medium show higher starvation resistance than flies developed on protein enriched medium. Greater starvation resistance requires physiological changes which are likely to trade-off with other fitness-related traits. By reducing the amount of nutrition in particular protein (yeast) offered to adult flies (caloric restriction) increases their starvation resistance, with up to twofold difference between females previously fed *ad labium* yeast than those given no yeast [54], [55], [56], [57], [58]. Sisodia and Singh [26] also found that south Indian populations of *Drosophila ananassae*, which feed on carbohydrate rich fruits, have higher starvation resistance than north Indian populations which feed on protein enriched fruits. For starvation there is good evidence that an increase in the lipid content of adults underlies increased resistance to starvation.

There are several factors which contribute to starvation resistance although their general importance is uncertain. An increase in body weight has been associated with starvation resistance and body weight may reflect the total reserve of energy storage compounds carried by organisms. However, a reduced rate of respiration could underlie starvation resistance; there was no correlated change in respiration rate in lines selected for female starvation resistance [59]. There is also evidence for an association between starvation resistance and carbohydrate metabolic reserves, particularly as the association between starvation and energy reserves is strongest when both carbohydrate and lipid components of these reserves are considered [60]. Our earlier results [26] suggest that flies from different localities differ in their susceptibility to starvation because of differences in their propensity to store body lipid.

### Cold Tolerance

Flies developed on carbohydrate rich diet have fast recovery than flies developed on protein rich diet. There are many possible physiological explanations for the faster recovery from chill-coma when flies are fed a carbohydrate-enriched diet. Carbohydrate is well known to increase the fat content of the flies [61]. There is positive correlation between body lipid content in *Drosophila* spp. and resistance to cold temperature [62] and starvation [63] and desiccation stress [64]. Sisodia and Singh [26] found evidence of positive correlation between lipid content and starvation resistance in *Drosophila ananassae.* Anderson et al. [48] also found that flies grown on carbohydrate–enriched medium have faster recovery from chill-coma than flies grown on protein-enriched medium in *Drosophila melanogaster.* Hence it is possible that the faster recovery from chill-coma of the flies raised on carbohydrate-enriched medium was due to larger lipid deposits. The physiological basis for how fat deposits improve chill coma recovery is not completely understood. Larger lipid deposits may affect the quality and/quantity of the cuticular hydrocarbons influencing water loss and update of the cell [65]. Protein –rich food provides an energy source that is more complicated to utilize as compared to a carbohydrate-rich diet. Sisodia and Singh [45] found that flies from higher latitudinal areas i.e. north Indian populations are more resistant to chill coma, consistent with the higher level of cold stress likely to be encountered at high latitudes, flies from low latitudinal area i.e. south Indian populations recovered from chill coma slowly than populations of north India, living at high latitudinal areas.

An increase in lipid reserves is also induced by protein-poor rich adult diet [57], [66]. This response presumably mediates at least part of the induced response of starvation resistance to caloric restriction. Hoffmann et al. [62] did not find any correlation between lipid reserves and starvation resistance among isofemale strains derived from wild populations, either within or across populations, whereas Baldal et al. [67] observed that raising larvae under crowded conditions increases the adult fat content without improving starvation resistance. Therefore, storing more reserves is a common adaptation to starvation in laboratory experiments but higher lipid content does not lead to greater starvation resistance.

### Heat Tolerance

We found that flies developed on protein enriched medium have higher heat resistance than flies grown on carbohydrate enriched medium. Very few flies developed on carbohydrate rich diet have revived after heat shock. Flies developed on protein rich diet cope up with heat shock faster than flies developed on carbohydrate –rich diet. The physiological explanation for an increased heat knockdown tolerance among flies developed on protein enriched medium is unknown. One possibility may be related to the induction of heat shock proteins which are known to be important for coping with several stress types [29], [68], [69], [70], [71], [72]. Anderson et al. [48] reported that Hsp 70 is upregulated in flies developed on protein enriched medium compared to in flies developed on protein deficient medium.

### Life History Traits

We found a higher females’ developmental success on protein-enriched medium while males’ developmental success was higher on carbohydrate enriched medium. This shows that two sexes have different requirements during development and growth. Our results are consistent with the findings of Anderson et al. [48] (2010) which prove that *Drosophila* spp. have similar type of sex specific requirements. Previous studies show that *Drosophila melanogaster* females accumulate more lipid but less protein relative to body mass compared to males [73], while females need to build protein for ovaries [17], [18] males accumulate protein to build up muscles mass for activity during courtship. Sex – specific responses in life-history traits are well-known from other studies on *Drosophila melanogaster* [74], [75], [76].

Egg production in females developed on protein enriched medium is higher than females developed on carbohydrate enriched medium. A high protein requirement when producing eggs might reflect that synthesis of the egg-yolk protein vitelline in females is dependent on the incorporation of amino acids [15], [17]. The interesting finding of this study is that flies evolving under protein rich condition had reduced egg to adult viability suggest a trade-off between egg to adult survival and egg production. This trade-off could suggest that a limiting shared resource is divided between the two traits. However, the trade-off was found on both diet types. Thus it is more likely that the trade-off is caused by antagonistic pleiotropy. Kristensen et al. [76] found trade-off between egg to adult survival and body mass in protein rich diet in *Drosophila melanogaster.* They also explained that this event is caused by antagonistic pleiotropy, whereby alleles coding for larger body size which is advantageous under protein-enriched conditions, at the same time have a negative effect on physiological processes that affect survival. This result can be extrapolated to other organisms including humans. It introduces interesting challenges and potentials in relation to breeding strategies and diet recommendation. Furthermore, results from this experiment indicate that trade-off between fitness traits may exist when dietary protein content is varied. This may potentially have consequences for populations (including human populations) that in recent times have changed their diet fundamentally. Our data suggest that such a change may simply provide an immediate challenge to the generations exposed to the change. Evolutionary adaptation to the new diet may potentially produce an additional risk diet through unfavorable trade-offs.

We show many surprising differences in stress adaptation, life history traits and reproduction between flies developed on two different nutritional regimes. These data raise issues about the role of diet and specifically the dietary Protein: Carbohydrate ratio in maintaining variation for these traits within and among population. So, the ability to use different food sources is likely to be under strong selection if organisms are faced with natural variation in macro-nutrients (protein, carbohydrate and lipid) availabilities.

Thus, our results that nutrition affects resistance towards a variety of stress types in *Drosophila ananassae* is interesting in an ecological, an evolutionary as well as in a physiological context. The quality of diet at larval stage plays a crucial role in flies’ responses to cope with different challenges such as reproduction, survivorship and stress resistance. This indicates that selection pressure on the ability to handle these tasks will interact with the nutritional conditions.
